# The RADx Tech Clinical Studies Core: A Model for Academic Based Clinical Studies

**DOI:** 10.1109/OJEMB.2021.3070830

**Published:** 2021-04-28

**Authors:** Laura L. Gibson, Nisha M. Fahey, Nathaniel Hafer, Bryan Buchholz, Denise R. Dunlap, Robert L. Murphy, Chad Achenbach, Cheryl Stone, Rebecca Cleeton, Jared O’Neal, Jennifer K. Frediani, Miriam B. Vos, Oliver Brand, Risha Nayee, Leona Wells, Wilbur A. Lam, Greg S. Martin, Yukari C. Manabe, Matthew L. Robinson, John P. Broach, Jeffrey E. Olgin, Bruce Barton, Stephenie C. Lemon, Allison Blodgett, David D. McManus

**Affiliations:** University of Massachusetts Medical School, Worcester, MA 01655 USA; University of Massachusetts Medical School, Worcester, MA 01655 USA; University of Massachusetts Medical School, Worcester, MA 01655 USA; University of Massachusetts Lowell, Lowell, MA 01854 USA; University of Massachusetts Lowell, Lowell, MA 01854 USA; Northwestern University Feinberg School of Medicine, Chicago, IL 60611 USA; Northwestern University Feinberg School of Medicine, Chicago, IL 60611 USA; Children’s Healthcare of Atlanta Inc, Atlanta, GA 30322 USA; Children’s Healthcare of Atlanta Inc, Atlanta, GA 30322 USA; Emory University School of Medicine, Atlanta, GA 30322 USA; Emory University Nell Hodgson Woodruff School of Nursing, Atlanta, GA 30322 USA; Emory University School of Medicine, Atlanta, GA 30322 USA; Georgia Institute of Technology, Atlanta, GA 30332 USA; Emory University School of Medicine, Atlanta, GA 30322 USA; Emory University School of Medicine, Atlanta, GA 30322 USA; Emory University School of Medicine, Atlanta, GA 30322 USA; Emory University School of Medicine, Atlanta, GA 30322 USA; Johns Hopkins University School of Medicine, Baltimore, MD 21205 USA; Johns Hopkins University School of Medicine, Baltimore, MD 21205 USA; University of Massachusetts Medical School, Worcester, MA 01655 USA; University of California San Francisco, San Francisco, CA 94158 USA; University of Massachusetts Medical School, Worcester, MA 01655 USA; University of Massachusetts Medical School, Worcester, MA 01655 USA; University of Massachusetts Medical School, Worcester, MA 01655 USA; University of Massachusetts Medical School, Worcester, MA 01655 USA

**Keywords:** SARS-CoV-2, COVID-19, *in vitro* diagnostics, point-of-care testing, rapid acceleration of diagnostics

## Abstract

The National Institutes of Health (NIH) launched the Rapid Acceleration of Diagnostics (RADx^SM^) Tech initiative to support the development and commercialization of novel severe acute respiratory syndrome coronavirus 2 (SARS-CoV-2) point-of-care test devices. The primary objective of the Clinical Studies Core (CSC) was to perform SARS-CoV-2 device studies involving diverse populations and settings. Within a few months, the infrastructure for clinical studies was developed, including a master protocol, digital study platform, data management system, single IRB, and multi-site partnerships. Data from some studies are being used to support Emergency Use Authorization of novel SARS-CoV-2 test devices. The CSC reduced the typical time and cost of developing medical devices and highlighted the impactful role of academic and NIH partnership in addressing public health needs at a rapid pace during a global pandemic. The structure, deployment, and lessons learned from this experience are widely applicable to future in vitro diagnostic device clinical studies.

## I. INTRODUCTION

The World experienced the emergence of a novel coronavirus in December 2019 in central China, which was declared a global pandemic by the World Health Organization in March 2020. The International Committee on Taxonomy of Viruses named this virus severe acute respiratory syndrome coronavirus 2 (SARS-CoV-2), and the World Health Organization assigned the term coronavirus disease 2019 (COVID-19) to the lower respiratory tract disease caused by SARS-CoV-2. The first case of COVID-19 in the United States was diagnosed in January 2020, shortly after which the U.S. Department of Health and Human Services declared a public health emergency. SARS-CoV-2 spread rapidly throughout the U.S., soon exceeding public health laboratory capacity to keep pace with the demand for diagnostic testing. The Food and Drug Administration (FDA) authorized a test developed by the Centers for Disease Control and Prevention (CDC) via an Emergency Use Authorization (EUA) on February 4, 2020, which permitted use of a non–FDA-approved test device to respond to an emergency.

Mitigating strategies to reduce propagation of the pandemic centered on wearing masks, social distancing, and timely testing [1], [2]. However, the United States had insufficient capacity for rapid SARS-CoV-2 testing that would reduce public health risk and elucidate the true burden of infection [3]–[5]. Consequently, the National Institutes of Health (NIH) received an appropriation from Congress in April 2020 to increase the United States capacity to conduct SARS-CoV-2 viral antigen and nucleic acid testing [6]. In an effort to support the development, validation, and commercialization of these tests, the NIH launched the Rapid Acceleration of Diagnostics (RADx^SM^) Tech initiative and challenged scientists to develop rapid SARS-CoV-2 diagnostic testing and screening technologies that could be deployed across the country in diverse populations and settings [7].

Here we describe creation of the RADx Tech Clinical Studies Core (CSC) by leveraging existing infrastructure and multidisciplinary expertise from leading academic healthcare centers across the United States. We also illustrate the principles of team science and coordination that grounded our approach.

## II. Objectives

The primary objective of the CSC was to design and implement SARS-CoV-2 diagnostic device clinical studies involving diverse use-case populations and settings.

COVID-19 has affected segments of the U.S. population that have traditionally experienced disparities across a myriad of health conditions [8]–[10]. Priority populations for clinical studies were thus communities that have and will likely continue to experience a high burden of COVID-19, such as Native American/American Indian populations, African Americans, Latinx communities, certain Asian groups, and others who have borne a disproportionate share of the morbidity and mortality attributed to COVID-19 [10]–[16]. In addition, urban areas of the U.S. were hit hardest early in the pandemic, while more recently, rates of COVID-19 are increasing in many rural areas across the country [17], [18]. Regardless of geography or socio-economic status, those in congregate facilities such as nursing homes or prisons have experienced particularly high rates of COVID-19 mortality [16], [19], [20]. Inclusion of multiple research sites across the U.S. allowed the CSC to draw on the relationships between each institution and its local communities to maximize opportunities for study enrollment.

In addition, SARS-CoV-2 diagnostic technologies need to be rigorously evaluated not only for technical performance characteristics, but also in the context of “real-world” use. The entire testing workflow must be evaluated for its usability in the populations and settings for which it is intended. Moreover, a mechanism for reporting and disseminating test results need to be established for both individual and public health purposes. Providing these resources, the RADx Tech CSC highlights the impactful role of academic and NIH partnership in addressing public health needs at a rapid pace during a global pandemic. The structure, deployment, and lessons learned from this experience are widely applicable to future medical technology clinical studies.

## III. Approach

### A. Existing Infrastructure

First established in 2009 by NIH National Institute of Biomedical Imaging and Bioengineering (NIBIB) and now supported by multiple NIH Institutes and Offices lead by NIBIB, the Point-of-Care Technologies Research Network (POCTRN) was well-positioned to apply its mission to drive the development of point-of-care diagnostics in the context of the SARS-CoV-2 pandemic [21]. The RADx Tech CSC was developed by the Center for Advancing Point of Care Technologies (CAPCaT) in Heart, Lung, Blood, and Sleep Diseases, a POCTRN technology hub and an offshoot of a highly successful medical product incubator, the Massachusetts Medical Device Development Center (M2D2) at the University of Massachusetts Lowell (UML) and Medical School (UMMS) campuses [22]. Similarly pivoting from their respective areas of focus, other POCTRN centers at Northwestern (NU), Emory (EU), and Johns Hopkins (JHU) Universities were integral to CSC development and clinical study implementation. The Center for Innovation in Point-of-Care Technologies for HIV/AIDS at Northwestern University (C-THAN) provides expertise and support for development of diagnostic devices and assays used to screen, diagnose, and monitor HIV and its associated co-morbidities including viral hepatitis, tuberculosis, and cancers. While its network includes seven African academic partners, C-THAN has pivoted for RADx Tech to focus on Chicago with domestic partners Howard Brown Health Center and ACCESS Community Network. The Johns Hopkins Center for POC Technologies Research for Sexually Transmitted Diseases provided expertise developing sexually transmitted infections in vitro diagnostic devices from proof-of-concept to clinical performance evaluation (see Robinson et al, Clinical Review Committee in this issue). The Atlanta Center for Microsystems Engineered Point-of-Care Technologies (ACME POCT) is a disease-agnostic inter-institutional entity spanning Emory University, Georgia Institute of Technology, and Children’s Healthcare of Atlanta that focuses on the development and translation of microsystems-engineered technologies, and brought its unique combination of clinical and technical expertise to SARS-CoV-2 diagnostics. The NIH National Heart, Lung, and Blood Institute (NHLBI) provided oversight and funding for infrastructure, operations, and partnerships needed by the CSC to perform all clinical studies (U54HL143541–02S1 and U54HL143541–02S2).

### B. New Design

**Fig. 1.**
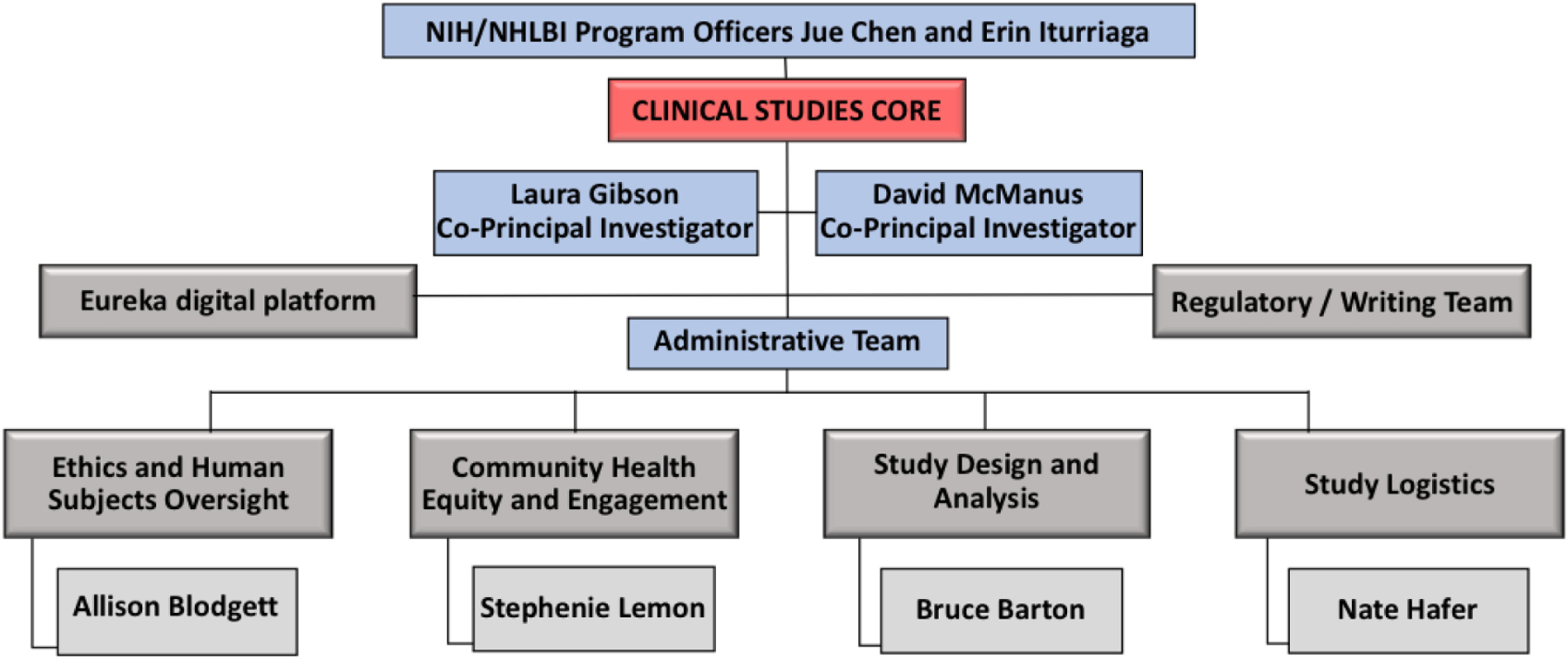
Clinical studies core organizational structure.

Applications for individual novel devices for SARS-CoV-2 detection progressed through the RADx Tech pipeline as described in other articles in this special issue, which included resources through the CSC to evaluate device performance and usability in prospective human studies. The CSC organizational framework consisted of two co-principal-investigators, an administrative team, and four other teams based on their roles in implementing clinical studies (Fig. 1). These teams were led by co-investigators with critical expertise in clinical study design, biostatistics, human subjects research, study logistics, and community engagement. Branding with the COVID-19 Test Us, COVID-19 Test Us Kids, and COVID-19 Test Us Bank names and logos provided consistent and recognizable signs of RADx Tech clinical studies.

The CSC **Study Design and Analysis** (**SDA**) **Team** developed the platform trial design in which multiple devices were studied in the context of a single disease in a perpetual manner. Because devices were ready for prospective clinical studies at different times and the duration of their studies may vary, this study design allowed devices to enter the platform at any time, and similarly to leave at any time upon successful completion of enrollment or for unsuccessful results identified on interim analysis. Use of a master protocol and consent, standard templates, and device and site-specific modular amendments provided flexibility to test a variety of technologies designed for multiple environments (e.g., self-administered test at home or clinician-administered point-of-care test in a medical office or skilled nursing facility), and allowed rapid Institutional Review Board (IRB) review and standardization across studies [23]. Throughout and at the end of device studies, the SDA Team monitored enrollment goals, data integrity, and device performance in real time. In addition, an independent Data and Safety Monitoring Board (DSMB) established by NHLBI had oversight of the device studies seeking EUA from the FDA, and met at least monthly to examine participant enrollment and safety, device performance, and provide recommendations to the NHLBI. The SDA Team generated detailed study reports for review by CSC leadership, the NIH, and DSMB based on datasets within the digital platform. They also harmonized these datasets for submission to the NIH COVID-19 RADx Data Hub, a centralized repository and analytical toolkit for de-identified SARS-CoV-2 testing data available to researchers (https://test.radx-hub.nih.gov/home).

All RADx Tech COVID-19 Test Us, Test Us Kids, and Test Us Bank studies were executed entirely through the NIH-supported Eureka **digital study platform** developed at the University of California San Francisco (UCSF) as a resource for mobile health research. The Eureka platform was selected based on its track record of conducting a variety of digital studies (currently 40 studies engaging over 400000 participants) and rapidly deploying a fully digital study within weeks. Rather than build a new mobile app for each study, participant-tested workflows are used to customize study activities that can be dynamically updated to the Eureka mobile app. Moreover, the Eureka team that runs the platform has expertise in the design and implementation of digital studies, which was leveraged for the rapid cycle deployment required for CSC device studies. The platform is available as a web-based interface and a mobile app (iOS and Android), is HIPAA compliant, runs on Amazon Web Services (AWS), and provides for scalability, redundancy, security, and data integrity. For CSC projects, participants engaged with Eureka to assess eligibility for a device study, provide consent, and complete digital surveys. An initial survey contained questions about demographics, substance use, medical conditions, and COVID-19 experience including infection history, symptoms, treatment, and vaccination status. After samples were collected, a second survey asked about result interpretation and device usability. All study steps within Eureka were supported by a messaging system utilizing email, SMS, and/or push notification. Research staff managed their site participants through a secure study management portal, including a collection of triggerable digital case report forms that open and close with time windows used to enter device test results and other participant-specific data.

**Fig. 2.**
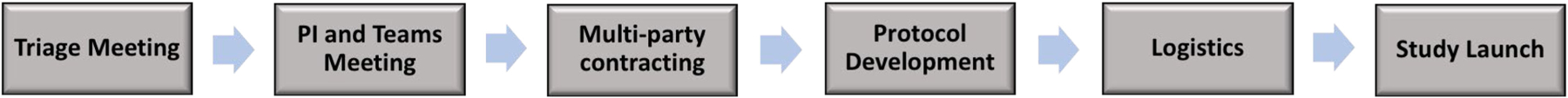
Clinical studies core project workflow.

The CSC workflow (Fig. 2) began with a 2-step intake process focused on understanding the regulatory goals and timeline of the company and the intended use and technical aspects of the device. Multi-party contracting proceeded in parallel. Based on this assessment, the writing team met daily to create device-specific protocols with the overall objective to test the performance (as positive percent agreement and negative percent agreement), usability, feasibility, and safety of the device. Every protocol included a comparator SARS-CoV-2 assay (performed via partnership with Quest Diagnostics in Marlborough, MA or a local site CLIA certified reference laboratory) and fulfilled other requirements for FDA EUA. Protocol development was a collaborative process with engagement from sponsors, the Eureka build team that customized workflows for each device study, regulatory consultants, NHLBI Program Officers, the DSMB, and study sites. Linked but discrete device and site protocol modules separated consistent from variable content thus streamlining review and modification, and allowed flexible site recruitment strategies appropriate for local testing demand and practices. Embedded in this process, the CSC **Ethics and Human Subjects Oversight (EHSO) Team** ensured that all study documents were written according to regulations for the conduct of human subjects research, advised on best practices, addressed any ethical challenges, and performed pre-review before submission to the IRB. The CSC single IRB was based at UMMS with reliance agreements with affiliate research center IRBs managed by the EHSO Team.

Study protocols, materials, and Eureka content were also developed with leadership and guidance from the **Community Health Equity and Engagement (CHEE) Team**, which was created so that novel SARS-CoV-2 tests would have a strong likelihood of usability and acceptability across diverse populations and settings throughout the United States. The CHEE Team ensured CSC commitment to diversity, inclusivity, and patient-centered research. Given its broad charge, the CHEE Team assembled a two-fold approach that included six faculty members from the University of Massachusetts Schools of Medicine and Nursing who have scientific and/or clinical expertise working with CSC priority populations and settings as well as health equity and community engagement more generally. Activities lead by the CHEE Team included reviewing all CSC study documents for appropriate literacy levels, measures, and materials, developing a culturally representative website, outreach, and recruitment tools, making all participant interfaces available in both English and Spanish, and creating dissemination resources for CSC study participants.

In parallel with development of study protocols, the **Study Logistics Team** managed all aspects of study preparation and implementation. CSC device studies were conducted in partnership with a robust network of research sites, thus ensuring ethnically, geographically, and economically diverse study cohorts. The academic centers of POCTRN – UMMS, Northwestern, Johns Hopkins, and Emory Universities – each conduct research at a variety of sub-sites. In addition, the Agency for Healthcare Research and Quality-affiliated Practice-Based Research Network (PBRN) includes groups of primary care practices and clinicians who collaborate to conduct applied research projects and quality improvement initiatives, and to translate evidence into practice in order to address local urban and rural community health challenges. PBRNs span the country and largely serve populations that experience health disparities, including racial and ethnic minority populations. In particular, Oregon Rural Practice-Based Research Network, Kansas Patients and Providers Engaged in Prevention Research, Iowa Research Network, and the Southeast Regional Clinicians’ Network / Morehouse School of Medicine collaborated with the CSC as study sites. The Logistics Team established necessary legal agreements between UMMS and research centers, followed by extensive site onboarding that included review of standard operating procedures and training on the Eureka platform. They tracked and ensured timely shipping and receiving of devices, created research staff workflows for participant enrollment and sample collection, interfaced with sites on study implementation, and supported many other activities critical to the successful completion of device studies.

### C. Clinical Studies

While most clinical studies were designed to support EUA for novel SARS-CoV-2 diagnostic devices based on FDA requirements, the CSC is also pursuing other types of studies.

To support future research, a curated biorepository called COVID-19 Test Us Bank is being launched at JHU and UMMS to store and catalogue clinical samples obtained from participants who also complete the digital survey. For example, details about vaccination status, COVID-19 treatment, and SARS-CoV-2 infection history of Test Us Bank sample donors would be valuable for understanding infectivity or transmission of viral variants in these populations. This biorepository will offer value to SARS-CoV-2 investigators and RADx Tech-supported companies looking to use banked specimens for EUA.

The CSC also launched a study to support development of a novel approach to SARS-CoV-2 home testing called COVID-19 Test at Home. The study evaluated the feasibility of home testing by pairing an application created by the software developer CareEvolution and the QuickVue SARS Antigen Test developed by the diagnostics manufacturer Quidel. The app is intended to instruct participants on appropriate use of the test and facilitate correct interpretation of results by allowing participants to upload smartphone images of the test strips to the CSC database, thereby simulating an approach that might be used by a healthcare system or public health office to track results. The findings of this study may inform development of digital platforms to support clinical and research programs for SARS-CoV-2 population-based home testing.

## IV. Lessons Learned

The CSC encountered many challenges and opportunities for innovation. The salient lessons learned are summarized in Table I. Given the rapid timeline, the infrastructure and operations framework for the CSC itself were developed simultaneously with clinical study protocols and collaborations with study sites. Moreover, critical elements common to all functional areas included communication and coordination across CSC and external teams. Regular and often concurrent multidisciplinary meetings spanning 7 days a week were required of the CSC teams and staff, the Eureka team, NHLBI Program Officers, and device companies. A central accessible document repository allowed secure sharing according to roles and needs. Balancing comprehensiveness with speed was a daily endeavor and guided all decisions as advancement and redirection occurred in parallel, and progress was made iteratively based on accumulating knowledge and experience. At the same time, SARS-CoV-2 national priorities, governmental agency guidelines on sampling and testing strategies, COVID-19 clinical management, and understanding of SARS-CoV-2 pathogenesis (e.g., viral dynamics or emergence of viral variants) were co-evolving with RADx Tech and the CSC, which added multi-faceted complexity. While keeping their focus on ending the SARS-CoV-2 pandemic, CSC members together developed a culture of mutual respect, patience, support, and professionalism that has persisted over the course of RADx Tech clinical studies.

Our teams learned to expect change, at times by the hour, and this unpredictability drove new approaches and higher levels of efficiency. Leveraging existing resources and partnering with ongoing initiatives were often the most effective. For example, the Commonwealth of Massachusetts created the “Stop the Spread” (STS) program to provide low barrier, free of cost, and easy to access SARS-CoV-2 testing to all state residents (https://www.mass.gov/info-details/stop-the-spread). As the largest healthcare provider in central MA, the UMass Memorial Healthcare (UMMHC) system joined STS to host and staff community testing events in neighborhoods throughout Worcester, MA. Based at UMMS on the medical center campus, the CSC partnered with UMMHC to stage clinical study recruitment stations at community events. Attendees could receive their SARS-CoV-2 test as part of the event or by enrolling in a device study. Similar recruitment strategies were undertaken at the clinical research center, ambulatory clinics, and inpatient units at the medical center. Tasked with accommodating a variety of novel devices, anticipating technical needs (including sufficient power sources at a myriad of outdoor community event settings), and ensuring appropriate sample transport, the Logistics Team and research coordinators were vital contributors to study operations.

While existing resources were particularly useful, the relentless pandemic and the increasing demand for testing and other SARS-CoV-2 research required anticipating needs and creating new assets. For example, the standardized digital survey questions in Eureka for study participants required not only updating but also alignment with the NIH COVID-19 RADx Data Hub. Questions about vaccination and treatments were recently added to the survey to reflect trends in COVID-19 prevention and clinical management.

## V. Conclusion

The RADx Tech Clinical Studies Core was charged with testing SARS-CoV-2 diagnostic technologies in the diverse populations and settings in which they would be used. Within only a few months, the infrastructure for these studies was developed, including a platform study design with single IRB and standard documents, a digital study platform and data management system, mechanisms for community engagement, and multi-site collaboration. As part of the NIH RADx Tech initiative, the Clinical Studies Core reduced the typical time and cost of developing medical devices, and highlighted the impactful role of academic and NIH partnership in addressing public health needs at a rapid pace during a global pandemic [24]. The structure, deployment, and lessons learned from this experience are widely applicable to future in vitro diagnostic device clinical studies.

## Figures and Tables

**TABLE I:** Clinical Studies Core Lessons Learned

Maintain a balance comprehensiveness and speed to guide all decisions
Utilize unexpected change and unpredictability to drive new approaches and enhance efficiency
Develop infrastructure, operation, clinical study protocols, and collaboration with study sites simultaneously
Make iterative progress based on accumulation of knowledge and experience
Leverage existing resources and partner with ongoing initiatives
Conduct regular multidisciplinary meetings to enhance internal and external communication
Create a central accessible document repository for secure sharing according to roles and needs
Foster a culture of mutual respect, patience, support, and professionalism

## Impact Statement—

The Clinical Studies Core provided the infrastructure and expertise to rapidly design and implement clinical studies for novel SARS-CoV-2 diagnostic devices in the RADx Tech pipeline.
